# Secale Cornutum

**Published:** 1826-07

**Authors:** 


8. *Secale Cornutum.* Mr. Clark, surgeon, in Bristol, has related three cases of tardy labour and deficient pains where a scruple of the powdered ergot of rye brought on quick delivery. In one case only out of three, was the child dead.—*Med. and Phys. Journal, January.*

